# Supplementary material for: Identification of key genes as potential diagnostic biomarkers in sepsis by bioinformatics analysis
Source: PeerJ. 2024 Jun 18;12:e17542. doi: 10.7717/peerj.17542 (PMC11192024; doi:10.7717/peerj.17542)
Supplement: Supplemental Information 5 [file peerj-12-17542-s005.docx]

Dear Reviewer and Reviewers,

We sincerely apologize for any inconvenience caused by our work.

Youjie Zeng began contributing to our project starting from the revision on November 26, 2023. Prior to that, he was not involved in our work and therefore was not included in our list of authors. The reviewers provided us with meaningful suggestions, but we encountered some challenges in addressing them. Therefore, we sought assistance from Youjie Zeng, who made significant contributions to our newly revised manuscript, primarily in experimental design, data analysis, and providing critical revisions on our content. Specifically, his contributions are as follows:

Experimental methods: Methods sections 2.2, 2.6 and 2.9 were written by Youjie Zeng.

Bioinformatics analysis: The data analysis in Figure 2, Figure 6, and Figure 9 was conducted by Youjie Zeng.

Please feel free to contact us if you have any questions or concerns.
